# Dynamic Cerebral Autoregulation in Embolic Stroke of Undetermined Source

**DOI:** 10.3389/fphys.2020.557408

**Published:** 2020-10-20

**Authors:** Hongyin Ma, Jia Liu, Shan Lv, Peng Zhang, Wei-Tong Guo, Yang Qu, Zhen-Ni Guo, Yi Yang

**Affiliations:** ^1^Stroke Center, Department of Neurology, The First Hospital of Jilin University, Changchun, China; ^2^Shenzhen Institutes of Advanced Technology, Chinese Academy of Sciences, Shenzhen, China; ^3^Neuroscience Center, Department of Neurology, The First Hospital of Jilin University, Changchun, China

**Keywords:** cerebral autoregulation, vascular function, transcranial Doppler, transfer function analysis, ischemic stroke

## Abstract

**Background and Purpose:**

Dynamic cerebral autoregulation (dCA) in acute ischemic stroke is probably compromised. Although the characteristics of dCA in different types of stroke have been largely investigated, dCA in embolic stroke of undetermined source (ESUS) remains poorly understood. In this group, we aimed to elucidate the characteristics of dCA and their relevance to clinical outcomes.

**Methods:**

The study enrolled 77 ESUS patients and 50 controls. Bilateral cerebral blood flow velocities (CBFV) of middle cerebral arteries and arterial blood pressure were simultaneously recorded using a transcranial Doppler combined with a servo-controlled finger plethysmograph. Transfer function analysis was used to obtain dCA parameters including phase, gain, coherence at very low frequency (VLF) and low frequency (LF), and the rate of recovery (RoRc) of CBFV. A multivariable logistic regression model was established to explore the relationship between dCA and clinical outcomes.

**Results:**

Gain at VLF and LF, phase at LF, and RoRc of CBFV in bilateral hemispheres of the ESUS group were consistently worse than those of the control group (all *P* < 0.001). Bilateral RoRc of CBFV was significantly higher in patients with favorable outcomes than in those with unfavorable outcomes (stroke hemisphere: *P* < 0.001; non-stroke hemisphere, *P* = 0.029). Rate of recovery of CBFV in stroke hemisphere >13.3%/s was an independent predictor of favorable clinical outcomes (adjusted odds ratio = 30.95, 95% CI: 5.33–179.81, *P* < 0.001).

**Conclusions:**

Dynamic cerebral autoregulation was relatively impaired in both stroke and non-stroke hemispheres in ESUS patients, and functioning dCA after ESUS may indicate favorable clinical outcomes.

## Introduction

Cerebral autoregulation (CA) maintains cerebral blood flow (CBF) at an approximately constant level within a certain range of arterial blood pressure (ABP) fluctuations (Lassen, 1959). The use of transcranial Doppler (TCD) in clinical practice has led to the gradual acceptance of dynamic cerebral autoregulation (dCA), which characterizes the temporal capacity of CBF recovery following a sudden change in ABP (Aaslid et al., 1989). As reduced effectiveness of autoregulation renders the brain more sensitive to both hypo- and hyperperfusion, dCA measurements are of significance in a variety of pathological settings, particularly in stroke patients. Dynamic cerebral autoregulation in acute ischemic stroke (AIS) patients is impaired to a certain degree, and the evidence accumulated over recent years suggests that dCA enables good prediction of clinical outcomes (Castro et al., 2017; Chi et al., 2018; Ma et al., 2018; Intharakham et al., 2019). Due to distinct pathological changes, the characteristics of dCA in different stroke subtypes may vary. Among all stroke subtypes, research into large artery atherosclerosis and small artery occlusion subtypes has been of substantial focus (Immink et al., 2005; Guo et al., 2014, 2015; Petersen et al., 2015). Nevertheless, the underlying pathogenesis of stroke with undetermined cause (cryptogenic stroke) has remained elusive, and dCA characteristics of this stroke subtype remain underexplored.

As a typical subset of cryptogenic stroke, embolic stroke of undetermined source (ESUS) has been proposed as a non-lacunar brain infarct without proximal arterial stenosis or cardioembolic sources. This concept was originally put forward in 2014 as a therapeutically relevant entity based on evidence that most cryptogenic strokes were embolic (Hart et al., 2014). In fact, ESUS patients comprise, on average, 17% of all ischemic strokes (Hart et al., 2017). To the best of our knowledge, the characteristics of dCA in cryptogenic stroke or ESUS remain underexplored. A preliminary dCA study of the cryptogenic stroke population indicated that dCA was compromised only in the non-affected hemisphere (Tutaj et al., 2014). Furthermore, although ESUS patients may be included in some dCA studies of AIS, this subgroup has never been specifically analyzed (Saeed et al., 2013; Llwyd et al., 2018; Ma et al., 2018). In our previous dCA study of AIS patients (Ma et al., 2018), eight ESUS patients were enrolled. Compared to healthy controls, ESUS patients tended to exhibit bilateral impairment of dCA, but the generalizability of these findings and specific autoregulatory characteristics require further confirmation. Extending our dCA observations of ESUS patients may contribute to deeper understanding of stroke pathogenesis and provide individualized information to optimize therapeutic strategies in the future.

Transfer function analysis (TFA) is one of the most popular approaches to quantify dCA. It models dCA as a linear control system, where ABP is considered as the input and cerebral blood flow velocity (CBFV) as the output of the system, which maintains CBFV at a relatively constant level despite changes in ABP. The autoregulatory parameters, including phase, gain, and coherence in the frequency domain, can be derived from TFA to characterize dCA. In particular, large phase indicates that CBFV does not follow the changes of ABP (normal dCA), whereas small phase suggests impaired dCA. Gain can be used to quantify the change in magnitudes. Coherence is a metric of linearity between ABP and CBFV. When coherence is low, the assumption of linearity between ABP and CBFV is violated and the data needs to be discarded. In the time domain, the step response of CBFV indicates the recovery of CBF when a stepwise change in ABP occurs. A low rate of recovery (RoRc) of CBFV reflects a slow recovery of CBFV, which also indicates that the autoregulation is impaired.

Therefore, this study aimed to elucidate the characteristics of dCA in ESUS patients and their relevance to clinical outcomes.

## Materials and Methods

### Patients and Controls

This prospective observational study was performed at the Comprehensive Stroke Center, Department of Neurology, First Hospital of Jinlin University, China, from October 2017 to February 2019. This study complied with the Declaration of Helsinki, and ethical approval for the study was obtained from the Ethics Committee of the First Hospital of Jilin University, China (No. 2017-448). Written informed consent was obtained from all subjects or their direct relatives. Embolic stroke of undetermined source patients were diagnosed according to the criteria and protocol proposed by Hart et al. (2014). A series of diagnostic assessments were routinely arranged including brain computed tomography and brain magnetic resonance imaging, 12-lead electrocardiogram, Holter monitoring lasting for 24 h, transthoracic echocardiography, and extracranial and intracranial vascular imaging evaluations (typically TCD, brain magnetic resonance angiography, and carotid ultrasound). In addition to ESUS diagnosis, patients were required to satisfy the following criteria: (1) admitted to hospital within 7 days after stroke onset; (2) brain infarction involved in anterior circulation territory; (3) modified Rankin Scale (mRS) score of 0 prior to stroke; (4) sufficient bilateral temporal bone window for TCD insonation; and (5) conscious and could fully cooperate with dCA measurement. Patients with (1) a history of stroke within 3 months, (2) more than 50% stenosis or occlusion of intracranial and/or extracranial major artery in the non-stroke hemisphere, and (3) myocardial infarction, heart failure, severe anemia, and hyperthyroidism were excluded from the study.

In total, 50 age- and sex-matched volunteers without a history of stroke were enrolled as a control group. Control subjects underwent TCD and carotid ultrasound before dCA measurements to exclude asymptomatic intracranial and/or extracranial artery stenosis/occlusion.

### Clinical Data

All patients received routine stroke treatment and intensive nursing care in the stroke unit by the same clinical team in accordance with early stroke management guidelines (Powers et al., 2018). Demographic information, disease history, and laboratory tests of risk factors for stroke were recorded. Neurological examination and National Institutes of Health Stroke Scale (NIHSS) scores were evaluated in a timely manner at admission and discharge for each patient. Neuroimaging information was scanned, and the characteristics of stroke lesions were classified manually by two senior neuroradiologists. Functional outcomes were evaluated using mRS at 3 months after stroke onset. Favorable outcomes were defined as mRS scores <3 at 3 months.

### DCA Measurements

Dynamic cerebral autoregulation measurements were performed after the diagnosis of ESUS was established, and each patient was measured once during hospitalization, within 3–10 days after stroke onset. The measurements were performed in an exclusive laboratory room with minimal visual and acoustic stimulation. Room temperature was maintained at 22–24°C. Subjects were instructed to refrain from alcohol and caffeine intake and engaging in excessive exercise for at least 12 h before measurements. All measurements were performed by one specialized neurovascular ultrasound doctor. As preparation before measurements, subjects were instructed to rest in a relaxed supine position for 10 min. Baseline blood pressure was measured at the left brachial artery with an automatic blood pressure monitor (Omron 711, China). Bilateral middle cerebral arteries (MCA) were probed through temporal bone windows at a depth of 45–60 mm with 2-MHz probes applying TCD (MultiDop X2, DWL, Sipplingen, Germany) to record non-invasive CBFV. A servo-controlled plethysmograph (Finometer Pro, Netherlands) was used to continuously record spontaneous ABP. End tidal CO_2_ was measured using a capnograph with a face mask attached to the nasal cannula. After the ABP and CBFV signals were steadily obtained, real-time recording of measurements was initiated. During measurements, subjects were instructed to stay awake, breathe spontaneously, and minimize body movements for at least 5 min. After measurements, the data were stored in a personal computer for subsequent dCA analysis.

### DCA Analysis

Dynamic cerebral autoregulation analysis was performed using MATLAB (MathWorks, Natick, MA, United States). To eliminate possible time lags, beat-to-beat alignment of the data was achieved with a cross-correlation function. A third-order Butterworth low-pass filter (cutoff at 0.5 Hz) was applied as an anti-alias filter before down-sampling the data to 1 Hz. TFA was used to calculate the phase shift, gain, and coherence between ABP and CBFV at very low frequency (VLF, 0.02–0.07 Hz) and low frequency (LF, 0.07–0.20 Hz) bands (Zhang et al., 1998; Panerai, 2008) in the frequency domain. A value of coherence above 0.34 is considered valid for TFA (Claassen et al., 2016). In the time domain, the step response of CBFV denotes how fast the recovery of CBF is when a stepwise change in ABP occurs. To quantify the speed of recovery, RoRc of CBFV was defined as ΔCBFV/Δt × 100%, with the first 3 seconds being used for the calculation. According to a previous study (Aaslid et al., 1989), the reason for choosing a short interval of the step response is to fit it in a regression line, so as to quantify the cerebral vascular resistance (CVR) with its slope. A short interval from 1 to 3.5 s was chosen by Aaslid et al. (1989), because the regression line was in an approximately linear fashion within this range. In our previous study, we found that the first 3 seconds of the step response can be well approximated by a regression line, while a longer interval of the step response can violate the assumption of linearity between ABP and CBFV when the step response starts to converge to a plateau (Ma et al., 2016). Therefore, we used the first 3 seconds of the step response (CBFV following a step change of ABP) to calculate for the RoRc.

### Statistical Analyses

All data were analyzed using Statistical Program for Social Sciences version 21.0 (SPSS, IBM, West Grove, PA, United States). For continuous variables, normality of data was determined using the Kolmogorov–Smirnov test. Normally distributed data are expressed as mean ± SD, whereas non-normally distributed data are expressed as median (interquartile range). Categorical variables are reported by the rate or constituent ratio. The Wilcoxon signed-rank test was used to compare the dCA parameters between stroke and non-stroke hemispheres. The Mann–Whitney U test was used to compare the dCA parameters between ESUS patients and controls. The values of the bilateral dCA parameters in the control group were all used for comparison regardless of hemisphere. The chi-squared test was used to compare the differences between categorical variables. Univariate analyses were applied to identify correlations between clinical factors and dCA parameters. For continuous or ranked variables, Pearson/Spearman correlation was used. For two or multiple categorical variables, Mann–Whitney *U* test or Kruskal–Wallis H test was adopted for the univariate analyses. Repeated-measures ANOVA was used to compare the general trend of dCA parameter of stroke and non-stroke hemispheres between patients with favorable and unfavorable outcomes groups. A multivariable logistic regression model was established to identify the predictive value of the dCA parameter on clinical outcomes. Variables were selected according to clinical consideration and significant results of univariate analyses (*P* < 0.1) as reference. A receiver operating characteristic (ROC) curve was generated to determine the optimal cut-off point of dCA parameters associated with favorable/unfavorable clinical outcomes. Statistical tests were two-tailed. *P* < 0.05 was considered statistically significant.

## Results

**FIGURE 1:**
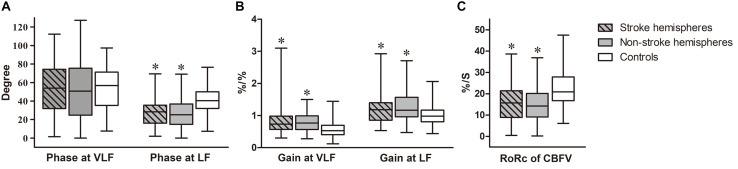
Autoregulatory parameters in ESUS patients compared to controls. Box-and-whisker plots of **(A)** Phase of stroke and non-stroke hemispheres in ESUS patients and controls, at very low frequency (VLF, 0.02–0.07 Hz) and low frequency (LF, 0.07–0.20 Hz) bands, respectively. **(B)** Gain of stroke and non-stroke hemispheres in ESUS patients and controls, at VLF and LF bands, respectively. **(C)** The rate of recovery of cerebral blood flow velocity (RoRc of CBFV) of stroke and non-stroke hemispheres in ESUS patients and controls. “^∗^” denotes *P* < 0.05 for comparison with controls (at corresponding frequency bands).

Measurements of 77 ESUS patients were performed with satisfactory signal quality a median of 6 days (range 3–10 days) after stroke onset. Measurements of 50 control subjects without a history of stroke were also collected. The demographic and clinical characteristics of subjects are presented in Table 1. There were no significant differences in age, sex, history of hypertension, diabetes mellitus, or current smoking status between ESUS patients and controls. Among ESUS patients, 12 had lesions in both left and right hemispheres; thus, there were 89 stroke and 65 non-stroke hemispheres in total. Physiological parameters during dCA measurements are summarized in Table 2. Excluding mean ABP, which was significantly higher in the ESUS group than in the control group (104.1 ± 13.1 vs. 89.2 ± 10.0, *P* < 0.001), other physiological parameters including heart rate, CBFV in both stroke and non-stroke hemispheres, and end tidal CO_2_ were not significantly different between ESUS and control groups. The main dCA parameters including phase, gain, and RoRc of CBFV of ESUS patients and controls are presented in Figure 1.

**TABLE 1:** Demographic and clinical information of participants.

	ESUS (*N* = 77)	Controls (*N* = 50)	*P* values
Age, years old	56.2 ± 10.3	53.2 ± 12.6	0.153
Sex, male	63 (81.8)	40(80.0)	0.798
Race			
Asian	77 (100)	50(100)	–
Current Smoking	40 (51.9)	21(42.0)	0.273
Hypertension	39 (50.6)	20(40.0)	0.240
Diabetes mellitus	19 (24.7)	10(20.0)	0.540
Previous symptomatic stroke	13 (16.9)	–	
Stroke lesion characteristics			
Lesion(s) on the left hemisphere	29 (37.7)		
Lesion(s) on the right hemisphere	36 (46.8)		
Lesion(s) on the both cerebral hemispheres	12 (15.6)		
Single acute lesion	40 (51.9)		
Multiple acute lesions	37 (48.1)		
Only cortical lesion(s) involvement	9 (11.7)		
Only subcortical lesion(s) involvement	51 (66.2)		
Both cortical and subcortical involvement	17 (22.1)		
Rt-PA thrombolysis	49 (63.6)		
NIHSS on admission	6 (4–9)		
NIHSS on discharge	2 (1–4)		
mRS at 3 months	1 (0–3)		
Laboratory results			
LDL-C, mmol/L	2.95 ± 0.8		
HbA1c, %	5.6 (5.4–6.5)		
Homocysteine, μmol/L	14.2 (10.6–26.1)		
Uric acid, mmol/L	333.5 ± 95.4		

*Data are expressed as mean ± SD, median (interquartile range) or absolute number (percentage) where appropriate. Rt-PA, recombinant tissue plasminogen activator; NIHSS, National Institutes of Health Stroke Scale; mRS, modified Rankin Scale; LDL-C, low density lipoprotein cholesterol; HbA1c, glycosylated hemoglobin A (1C).*

**TABLE 2:** Physiological data during dCA measurements.

	ESUS (*N* = 77)	Controls (*N* = 50)
MAP, mmHg	104.1 ± 13.1*	89.2 ± 10.0
HR, beats/min	68.0 (63.0–76.0)	68.0 (63.8–74.3)
Mean MCA CBFV, cm/s		
Stroke hemispheres	59.3 (48.0–79.3)	61.7 (56.4–68.5)
Non-stroke hemispheres	61.3 (52.2–72.8)	
EtCO_2_, mmHg	38.1 ± 1.4	38.0 ± 1.4

Data are expressed as mean ± SD or median (interquartile range) where appropriate. dCA, dynamic cerebral autoregulation; MAP, mean arterial blood pressure; HR, heart rate; MCA, middle cerebral artery; CBFV, cerebral blood flow velocity; EtCO_2_, end tidal CO_2_. *P < 0.05 compared with healthy controls.

### DCA Parameters in ESUS Patients vs. Controls

#### Phase

In the ESUS group, phase was not significantly different between stroke and non-stroke hemispheres at VLF and LF (VLF stroke vs. non-stroke hemispheres: 53.93 [31.96–74.26] vs. 50.69 [24.71–75.47], *P* = 0.31; LF stroke vs. non-stroke hemispheres: 28.42 [16.04–35.55] vs. 25.19 [14.77–36.84], *P* = 0.37). Compared to the control group (VLF: 56.77 [35.31–71.32]; LF: 40.45 [32.03–50.07]), the ESUS group had a lower phase in both stroke and non-stroke hemispheres at LF (both *P* < 0.001). This tendency was not significantly different at VLF bilaterally (stroke hemispheres, *P* = 0.60; non-stroke hemispheres, *P* = 0.27).

#### Gain

Gain at VLF and LF did not significantly differ between stroke and non-stroke hemispheres in ESUS patients (VLF stroke vs. non-stroke hemispheres: 0.74 [0.57–0.98] vs. 0.77 [0.56–0.99], *P* = 0.95; LF stroke vs. non-stroke hemispheres: 1.18 [0.86–1.40] vs. 1.16 [0.96–1.57], *P* = 0.82). The value of gain in both stroke and non-stroke hemispheres was significantly higher in the ESUS group than in the control group (VLF: 0.53 [0.40–0.70]; LF: 0.98 [0.81–1.17], all *P* < 0.001) at the corresponding frequency.

#### RoRc of CBFV

Rate of recovery of CBFV (%/S) was obtained to quantify the efficiency of step response. Consistently, this parameter in ESUS patients did not differ between stroke and non-stroke hemispheres (stroke vs. non-stroke hemispheres: 15.71 [8.91–21.41] vs. 14.25 [9.09–20.17], *P* = 0.14). The value of both hemispheres was significantly lower in the ESUS group than in the control group (20.91 [16.74–27.92], both *P* < 0.001).

### Clinical Factors Associated With DCA Parameters

Clinical factors of ESUS patients that correlated with dCA parameters were screened. Significant results are presented in Table 3. A small but consistent trend toward older age and poorer bilateral gain values at both VLF and LF was observed, although this did not reach statistical significance. Moreover, patients with a single stroke lesion tended to share better gain at LF than those with multiple stroke lesions. A higher uric acid level was associated with a poorer phase at LF bilaterally. Besides, sex, disease history, mean ABP, baseline NIHSS, whether patients underwent recombinant tissue plasminogen activator thrombolysis, stroke location, the time for dCA measurements, and other laboratory results were not correlated with any dCA parameter in both stroke and non-stroke hemispheres. In addition, there was no difference in dCA parameters between minor stroke (NIHSS ≤ 4) and moderate stroke (NIHSS > 4) patients (all *P* > 0.05) and no significant difference was found in dCA parameters between earlier (3–6 days) and later (7–10 days) measurements (all *P* > 0.05).

**TABLE 3:** Clinical factors of ESUS patients correlated with dCA parameters.

Clinical factors	dCA parameters	Hemisphere	Coefficient	*P* Value
Age	Gain at VLF	Stroke	0.219	0.055
Age	Gain at VLF	Non-stroke	0.203	0.105
Age	Gain at LF	Stroke	0.196	0.087
Age	Gain at LF	Non-stroke	0.242	0.052
Single/multiple stroke lesion(s)	Gain at LF	Stroke	—	0.093
Single/multiple stroke lesion(s)	Gain at LF	Non-stroke	—	0.002*
Uric acid	Phase at LF	Stroke	–0.318	0.005*
Uric acid	Phase at LF	Non-stroke	–0.290	0.019*
mRS at 3 months	Phase at VLF	Non-stroke	–0.254	0.041*
mRS at 3 months	RoRc of CBFV	Stroke	–0.246	0.031*

dCA, dynamic cerebral autoregulation; VLF, very low frequency; LF, low frequency; mRS, modified Rankin Scale; RoRc of CBFV, the rate of recovery of cerebral blood flow velocity. *P < 0.05 in according statistical analyses.

### DCA Parameters for Predicting Functional Outcomes

**FIGURE 2:**
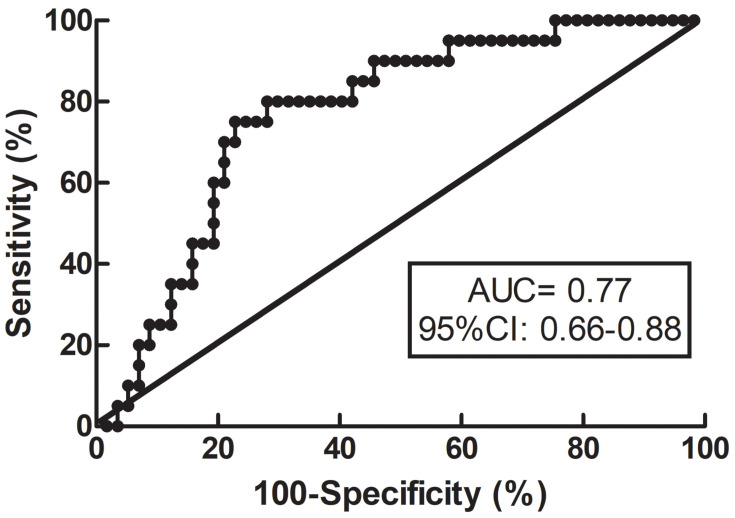
Receiver operating characteristic (ROC) curve for RoRc of CBFV. ROC curve for RoRc of CBFV in the stroke hemisphere was generated to calculate the cut-off value that optimized the sensitivity and specificity for predicting functional outcomes. RoRc of CBFV, the rate of recovery of cerebral blood flow velocity.

The median mRS score was 1 (interquartile range 0–3) at 3 months. In the ESUS group, 26% of the patients exhibited unfavorable outcomes. There was no significant difference in the time for measurements between patients with favorable and unfavorable outcomes (favorable outcomes, 6 [5–7]; unfavorable outcomes, 6 [4–7]; *P* = 0.586). Bilateral RoRc of CBFV in patients with favorable outcomes was significantly higher than that of patients with unfavorable outcomes (*P* = 0.013), and there was no interaction between side and outcome (*P* = 0.10). For other dCA parameters, there were no significant differences between patients with favorable outcomes and unfavorable outcomes. According to the results of the Spearman correlation, a higher RoRc of CBFV in the stroke hemisphere was correlated with lower mRS (*r* = −0.246, *P* = 0.031), indicating better functional outcomes, and a higher phase at VLF in the non-stroke hemisphere was correlated with lower mRS (*r* = −0.254, *P* = 0.041) (Table 3); no other significant correlation was detected between dCA parameters and mRS. Besides the dCA parameters, NIHSS at admission (*r* = 0.292, *P* = 0.010), NIHSS at discharge (*r* = 0.610, *P* < 0.001), and uric acid level (*r* = −0.268, *P* = 0.019) were associated with clinical outcomes in the ESUS group. In the subsequent regression model for predicting clinical outcomes, the independent variables including age, sex, time for dCA measurements, NIHSS at admission, uric acid level, and RoRc of CBFV on stroke hemisphere were involved. The optimal cut-off value of RoRc of CBFV on stroke hemisphere for predicting clinical outcomes was 13.3%/s (specificity of 77% and sensitivity of 75%). The ROC curve analysis suggested that the area under the curve (AUC) was 0.77 (95% CI: 0.66–0.88) (Figure 2). Rate of recovery of CBFV in the stroke hemisphere was dichotomized according to the optimal cut-off value. Multivariable logistic regression model revealed that RoRc of CBFV in the stroke hemisphere >13.3%/s was an independent predictor of favorable clinical outcomes (adjusted odds ratio = 30.95, 95% CI: 5.33–179.81, *P* < 0.001).

## Discussion

To our knowledge, this study is the first clinical dCA assessment focusing on ESUS patients. Our findings suggested that dCA was likely impaired in both stroke and non-stroke hemispheres after stroke onset. Further, dCA may be a valuable clinical prognostic marker in ESUS patients.

Embolic stroke of undetermined source patients comprise one in six of all ischemic stroke patients, with a relatively younger age of onset and annual recurrence rates averaging 4.5% per year, despite milder stroke severity (Perera et al., 2016; Hart et al., 2017). However, the specific pathogenesis and various emboligenic mechanisms of this subgroup have not been fully clarified. The impact of CA on its pathogenesis and clinical outcomes remains unknown. We selected the ESUS population rather than cryptogenic stroke population as the ESUS population possesses a clearer definition and diagnostic protocol for screening to ensure accurate interpretation of results. In the pioneering work of Tutaj, six patients with AIS of undetermined etiology were enrolled, and the lower phase of non-affected hemispheres compared to that of controls indicated that dCA was compromised in the non-affected hemisphere but not in the affected hemisphere (Tutaj et al., 2014). In our previous work on AIS, patients were classified according to Trial of ORG 10172 in Acute Stroke Treatment (TOAST) criteria (Adams et al., 1993), and eight patients had cryptogenic stroke and concurrently satisfied the diagnosis of ESUS. We observed that the impairment in dCA tended to have bilateral involvement according to the lower phase in both affected and non-affected hemispheres based on detailed data, but the small sample size limited the generalizability of these findings. Thus, this current study enrolled more ESUS patients to clarify the dCA characteristics. Similar to previous observations, dCA was bilaterally impaired as verified by multiple dCA parameters, including phase, gain, and RoRc of CBFV. In this regard, changes in all parameters were not significantly different between hemispheres. To ensure the robustness of our results, controls comprising age- and sex-matched volunteers with comparable concomitant vascular risk factors were enrolled. Although we are unable to perform dCA measurements prior to stroke for comparison, we surmise that acute stroke lesions are unlikely to play a major role in impaired dCA for the following reasons. First, stroke volumes of ESUS patients were relatively small and occasionally scattered; these focal lesions may not directly lead to evident dysautoregulation and are even more unlikely to compromise the non-stroke hemisphere. Moreover, despite ruling out large artery arteriosclerosis and small artery occlusion for current stroke etiology, the basis of cerebral vasculopathy already existed prior to stroke owing to the cardiovascular risk factor burden (Perera et al., 2016), which is thought to undermine the structural and functional cerebral vasculature and give rise to impaired dCA. This has been demonstrated by numerous clinical imaging findings of macroangiopathy of the carotid artery and evidence of microangiopathy, such as white matter hyperintensity and cerebral microbleeds, which are frequently detected in the ESUS population (Coutinho et al., 2016; Kashima et al., 2018; Komatsu et al., 2018; Kikuno et al., 2020). In addition, direct proof of vascular endothelial function impairment in ESUS patients was identified in a recent study by Shirai using flow-mediated vasodilation tests, suggesting that endothelial function was impaired in ESUS patients compared to that in controls (Shirai et al., 2020). Therefore, impaired dCA in ESUS patients may have existed prior to stroke onset and exerted underlying detrimental effects on cerebral hemodynamics, rendering brain tissue more vulnerable to the burden of emboli and resulting in hypo-perfusion and irreversible infarction (Higuchi et al., 2020).

The evidence accumulated over recent years strongly suggests that bilaterally impaired dCA has been identified in both large and small artery stroke (Dawson et al., 2003; Saeed et al., 2013; Guo et al., 2014, 2015; Panerai et al., 2015; Xiong et al., 2017; Intharakham et al., 2019). The main difference lies in that the impairment is probably more evident in the stroke hemisphere of large artery stoke compared to that in the non-stroke hemisphere. By contrast, differences in dCA impairment were not significant between hemispheres for small artery stroke. Relative to the substantial information available on other subtypes, little is known regarding the ESUS population. Our findings on the ESUS population provide a comprehensive characterization of dCA in AIS. Collectively, our results suggest that although differences in pathogenesis and vasculopathy may exist in various subtypes, dCA in AIS may be bilaterally compromised when considering subtypes. Additionally, although multiple dCA parameters including gain at the VLF and LF bands, phase at the LF band, and the RoRc of CBFV in the bilateral hemispheres of the ESUS group were consistently worse than those of the control group, indicating bilaterally reduced damping effect, slower recovery, and less effectiveness of dCA in the ESUS population, it seemed that the RoRc of CBFV was the most relevant and stable parameter for predicting clinical outcomes according to later univariate and multivariate analyses, especially for the RoRc of CBFV in the stroke hemisphere, in which an optimal cut-off value could be generated for predicting clinical outcomes with a moderate specificity and sensitivity. These results of RoRc were not conflicting with other dCA parameters in terms of arriving at a conclusion of impaired autoregulation in ESUS consistently. Moreover, the results also suggested a superior predictive value of the RoRc among the dCA parameters in the ESUS patients. We speculate that the RoRc may detect a more rapid response of CBFV than phase and gain, as the RoRc is calculated from the slope of the first 3 seconds of the step response. This suggests that the RoRc represents a dynamic relationship at the high frequency (HF) band (about 0.3 Hz) which is different from the frequency ranges where phase (normally at the LF band) and gain (normally at the VLF band) are sensitive. In our future research, more attention will be paid on this parameter to further assess its potential clinical relevance and value. With respect to the effects of dCA for predicting the clinical outcomes, previous research has provided ample evidence. In Castro’s study, dCA measurements were promptly performed within 6 h of ischemic stroke, and early effective autoregulation was proven to be associated with better neurological outcomes at 3 months (Castro et al., 2017). According to a recent study (Chi et al., 2018), mild AIS patients were enrolled for dCA assessments, and a predictive model was established. Their data revealed that phase <61° at VLF was independently associated with unfavorable outcomes. In agreement with these data, our previous observation of AIS patients indicated that the dCA parameters in the subacute stage were able to predict the clinical outcomes (Ma et al., 2018). Thus, combined with the results of our current study, dCA enabled good prediction of clinical outcomes applied to AIS and ESUS patients, and measuring dCA in clinical practice may be valuable for evaluating and establishing individualized therapeutic strategies.

This study has several limitations. First, due to restrictions of the TCD methodology, patients with poor temporal window penetration could not be measured successfully, which likely occurred in elderly female patients. Therefore, the composition of our study differed from the actual ESUS population, in which the proportion of men exceeds that of women; this may have generated a selection bias. Besides, given that cooperative and conscious patients were enrolled, the study more likely involved patients with milder stroke, which is another selection bias; thus, our findings cannot be generalized to severe stroke patients. Second, although we selected patients with stroke lesions involving the anterior circulation, some lesions may have involved the posterior circulation. The blood vessels for TCD monitoring are the bilateral MCA reflecting blood flow changes within the area of arterial blood supply; thus, dCA of the posterior circulation was not distinctively focused on in this study. Third, as dCA measurements were performed only once during the hospitalization within a wide range of 3–10 days after stroke onset, our data may not provide the characteristics of the temporal changes in dCA. Fourth, the optimal cut-off point was determined by generating an ROC curve and, then, dCA data were divided into two categories for further logistics regression analysis. This pre-specified cut-off value may lead to bias and should be considered when interpreting the data. Fifth, it should be noted that TCD was used to measure CBFV as a surrogate for CBF; thus, our results were based on the assumption that the vessel diameter of MCA remained constant over small physiological fluctuations, such as end tidal CO_2_ and ABP.

## Conclusion

In summary, dCA of ESUS patients was relatively impaired in both stroke and non-stroke hemispheres. Dynamic cerebral autoregulation may be a clinical prognostic marker in ESUS patients. As such, dCA measurements in clinical practice may be a useful research tool as well as a routine evaluation method for ESUS and other stroke patients.

## Data Availability Statement

The raw data supporting the conclusions of this article will be made available by the authors, without undue reservation.

## Ethics Statement

The studies involving human participants were reviewed and approved by the Ethics Committee of The First Hospital of Jilin University. The patients/participants provided their written informed consent to participate in this study.

## Author Contributions

HM, Z-NG, and YY designed the study. SL, W-TG, and YQ performed the data collection. HM, JL, and PZ performed the analyses. HM wrote the manuscript. All authors interpreted the data and had full access to the data and helped critically revise the manuscript before reviewing and approving the final version.

## Conflict of Interest

The authors declare that the research was conducted in the absence of any commercial or financial relationships that could be construed as a potential conflict of interest.
